# Digital Etch Technique for Forming Ultra-Scaled Germanium-Tin (Ge_**1**−*x*_Sn_*x*_) Fin Structure

**DOI:** 10.1038/s41598-017-01449-1

**Published:** 2017-05-12

**Authors:** Wei Wang, Dian Lei, Yuan Dong, Xiao Gong, Eng Soon Tok, Yee-Chia Yeo

**Affiliations:** 1 Department of Electrical and Computer Engineering, National University of Singapore, 117576 Singapore, Singapore; 2 Department of Physics, National University of Singapore, 117551 Singapore, Singapore

## Abstract

We developed a new digital etch process that allows precise etching of Germanium or Germanium-tin (Ge_1−*x*_Sn_*x*_) materials. The digital etch approach consists of Ge_1−*x*_Sn_*x*_ oxide formation by plasma oxidation and oxide removal in diluted hydrochloric acid at room temperature. The first step is a self-limiting process, as the thickness of oxide layer grows logarithmically with the oxidation time and saturates fast. Consistent etch rates in each cycle were found on the Ge_1−*x*_Sn_*x*_ samples, with the surfaces remaining smooth after etch. The digital etch process parameters were tuned to achieve various etch rates. By reducing the radio frequency power to 70 W, etch rate of sub-1.2 nm was obtained on a Ge_0.875_Sn_0.125_ sample. The digital etch process was employed to fabricate the Ge_1−*x*_Sn_*x*_ fin structures. Extremely scaled Ge_0.95_Sn_0.05_ fins with 5 nm fin width were realized. The side walls of the Ge_0.95_Sn_0.05_ fins are smooth, and no crystal damage can be observed. This technique provides an option to realize aggressively scaled nanostructure devices based on Ge_1−*x*_Sn_*x*_ materials with high-precision control.

## Introduction

Germanium-tin Ge_1−*x*_Sn_*x*_ has attracted significant research interest due to its potential applications in high-channel mobility metal-oxide-semiconductor field-effect transistors (MOSFETs)^1–4^ and silicon-based infrared photonic devices, e.g. near- and mid-infrared photodetectors^5–8^. In particular, recent theoretical and experimental studies indicate that strain-free Ge_1−*x*_Sn_*x*_ becomes a direct bandgap material at a Sn composition of about 6.5% to 11%^9–11^, making it a possible candidate as a gain medium in lasers based on group IV materials^12, 13^. Lasing of a direct bandgap Ge_0.874_Sn_0.126_ grown on Si substrates was reported recently^14, 15^.

Despite the large lattice mismatch between Ge and Sn (~15%) and the low equilibrium solid solubility of Sn in Ge (less than 1%), Ge_1−*x*_Sn_*x*_ with *x* up to 0.34 can be realized using non-equilibrium growth techniques such as molecular beam epitaxy (MBE) and chemical vapor deposition (CVD)^16–21^. As a group IV material, Ge_1−*x*_Sn_*x*_ can be integrated with Si-based complementary MOS (CMOS) technology. Bulk Ge_1−*x*_Sn_*x*_ n- and p- MOSFETs were reported recently with CMOS compatible processes^1–4^.

Ultra-scaled device architectures employing thin-body planar, nanowire or fin-type channels may be used in future CMOS transistors. A Fin-type field-effect transistor (FinFET) may employ Ge_1−*x*_Sn_*x*_ as a high mobility channel material, and methods to form Ge_1−*x*_Sn_*x*_ fins would be required. Formation of Ge_1−*x*_Sn_*x*_ nanostructures using conventional etches is often difficult as vertical or horizontal etch rates lack controllability in the absence of a stopping mechanism. To realize ultra-scaled Ge_1−*x*_Sn_*x*_ devices, a method to precisely etch Ge or Ge_1−*x*_Sn_*x*_ is highly desirable.

Digital etch is a high-precision etch technique that can provide tight control of etch variability. It has been studied for over 25 years on various materials, such as Si, Ge, III-Vs and some oxides^22–32^. There is a recent increase in interest in digital etch capabilities^33^. For Si and Ge, atomic layer etch has been demonstrated using either chlorine Cl or fluorine F adsorption that is subsequently followed by the removal of the halides by Ar ion bombardment^22–25^. For III-Vs, digital etch with etch rate of 1–3 nm/cycle has been reported using combinations of oxidation and oxide removal processes^26–29^. Recently, a two-step digital etch process consisting of a fluorine-based plasma etch followed by an acid wet etch was proposed to etch Ge_1−*x*_Sn_*x*_^34^. The formation of tin fluoride SnF_*y*_ inhibits further reactions between Ge_0.922_Sn_0.078_ and F radicals^35^. However, it is not an ideal self-limiting process due to the physical sputtering of the surface layer. The etch rate varies with plasma etch duration (1.5 nm/cycle and 3.2 nm/cycle for 10 s and 60 s etch duration, respectively)^34^. In addition, the formation of SnF_*y*_ is dependent on the initial Sn content, which limits its application for Ge_1−*x*_Sn_*x*_ with low Sn content.

Here we present a new digital etch process that allows the etching of Ge or Ge_1−*x*_Sn_*x*_ structures with sub-1.2 nm precision. The digital etch process is realized at room temperature, which avoid the Sn surface segregation or precipitation. The Ge_1−*x*_Sn_*x*_ surface after digital etch is smooth, and it can be used directly for extremely scaled Ge_1−*x*_Sn_*x*_ fin formation in Ge or Ge_1−*x*_Sn_*x*_ CMOS applications. In addition, such a unique process capability can also enable fabrication of Ge_1−*x*_Sn_*x*_ based nanostructures for a range of Si-compatible photonics and microelectro-mechanical (MEMS) device applications.

The Ge_1−*x*_Sn_*x*_ samples used for this study were grown on 4-inch Ge(100) substrates by a solid-source MBE system. The Ge substrates were cleaned by diluted hydrofluoric acid (DHF) before being loaded into the load-lock chamber of the MBE system. After transferring the substrates into the growth chamber, annealing was performed at 630 °C for 5 minutes to remove the native oxide. The substrate temperature was then adjusted to 100–150 °C for Ge_1−*x*_Sn_*x*_ growth. The thicknesses of the Ge_1−*x*_Sn_*x*_ layers are kept below 50 nm, so that all the Ge_1−*x*_Sn_*x*_ films are fully strained to the Ge substrates^20^. In addition, a commercial GeOI wafer was used to extract the etch rate of Ge.

**Figure 1:**
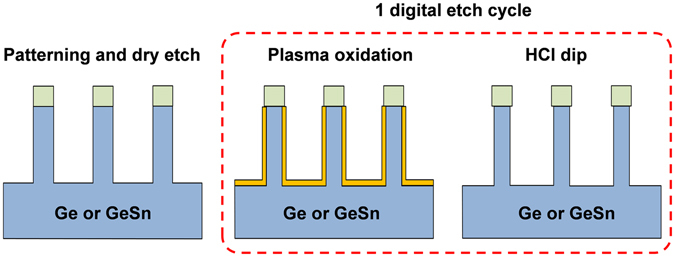
Illustration of the process flow for fabrication of extremely scaled Ge or Ge_1−*x*_Sn_*x*_ fins.

To avoid the problem caused by thermal instability of Ge_1−*x*_Sn_*x*_ layer having a high Sn content^36–38^, a low temperature process is preferred. Our digital etch approach forms a Ge_1−*x*_Sn_*x*_ oxide through exposure to oxygen plasma in an asher, and removes the oxide in dilute hydrochloric acid HCl (10%) for 30 s at room temperature. The plasma oxidation was performed at a pressure of 300 mTorr. As the oxidation reaction (first step) is self-limiting, the etch depth is no longer dependent on the etch time, but is dependent on the number of etching cycles. By repeating these two steps, the extremely scaled Ge or Ge_1−*x*_Sn_*x*_ fins can be formed in a controlled manner as shown schematically in Fig. 1.

## Ge_1−*x*_Sn_*x*_ Oxide Formation by Low Temperature Plasma Oxidation

It was reported that migration of Sn atoms from the Ge_1−*x*_Sn_*x*_ surface and from inside the Ge_1−*x*_Sn_*x*_ to the Ge_1−*x*_Sn_*x*_ oxide occurs during thermal oxidation^39^. Instead of using a conventional thermally activated oxidation at high temperature, a plasma activated oxidation is used as it can be done at low temperature. To better understand the mechanism of the digital etch process on Ge_1−*x*_Sn_*x*_, X-ray photoelectron spectroscopy (XPS) was performed to investigate the formation of Ge_1−*x*_Sn_*x*_ oxide during plasma oxidation, and removal of the surface oxide in HCl solution.

**Figure 2:**
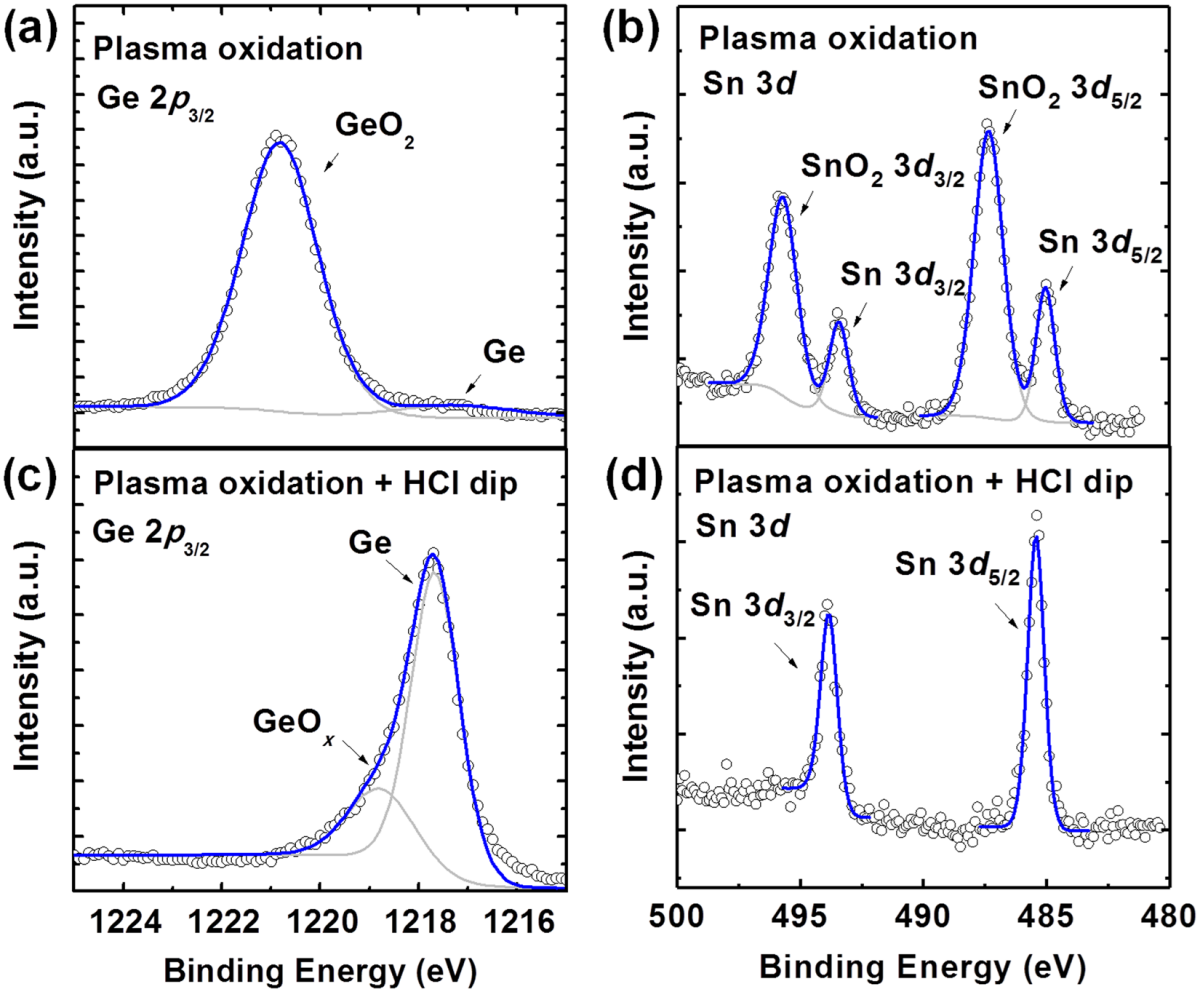
(**a**) Ge 2*p*_3/2_ and (**b**) Sn 3*d* XPS spectra of Ge_0.875_Sn_0.125_/Ge(001) sample after plasma oxidation. (**c**) Ge 2*p*_3/2_ and (**d**) Sn 3*d* XPS spectra of oxidized Ge_0.875_Sn_0.125_/Ge(001) sample after HCl dipping. XPS results indicate the oxide formation and removal in one digital etch cycle.

Figure 2a and b show the Ge 2p_3/2_ and Sn 3d photoelectron core spectra of the Ge_0.875_Sn_0.125_/Ge sample after plasma oxidation, with radio frequency (RF) power of 250 W and plasma oxidation time of 120 s. Both the peaks of the Ge and Sn oxides can be observed in the Ge 2p_3/2_ and Sn 3d photoelectron spectra, respectively. The binding energies (BEs) of these peaks agree well with the reported values of BEs for stoichiometric GeO_2_ and SnO_2_^39, 40^. As Ge 2p_3/2_ photoelectrons have a short inelastic mean free path λ (λ for Ge 2p_3/2_ is ~9.7 Å)^41^, no Ge peak can be observed in Fig. 2a, while clear Sn peaks can be identified in Fig. 2b. The core-level spectra were fitted using a combination of Gaussian and Lorentzian line shapes, together with a Shirley-typed background substraction. This results in the overall blue line fitting of the core-level spectra with their respective peak components (gray lines). The Sn content in the Ge_1−*x*_Sn_*x*_ oxide layer can be calculated using *A*_Sn-O_/(*A*_Sn-O_ + *A*_Ge-O_), where *A*_Sn-O_ and *A*_Ge-O_ are the normalized areas of the Sn-oxide and Ge-oxide peaks, respectively. We found that the Sn content *x* in the Ge_1−*x*_Sn_*x*_ oxide layer is 13.8%.

Figure 2c and d show the Ge 2p_3/2_ and Sn 3d spectra of the Ge_0.875_Sn_0.125_/Ge sample after plasma oxidation and HCl dipping. There were no obvious peaks related to Ge or Sn oxides in the Ge 2p_3/2_ and Sn 3d spectra, indicating that the Ge_1−*x*_Sn_*x*_ oxide formed by oxygen plasma can be effectively dissolved using a wet chemical treatment with dilute HCl solution. In Fig. 2c, a small shoulder can be observed besides Ge peak, which indicates a minor BE peak at 1218.9 eV after fitting. The peak is associated with native oxide on the Ge_1−*x*_Sn_*x*_ layer surface formed from atmospheric exposure before the *ex situ* XPS measurement. The smaller BE of the observed Ge oxide peak compared with that of stoichiometric GeO_2_ indicates suboxide GeO_*x*_ formation on the Ge_1−*x*_Sn_*x*_ layer owing to atmospheric exposure. The BEs of Sn 3d peaks slightly increases after HCl dip, which is attributed to the band bending originating from different charged states at the GeSn oxide/GeSn interfaces^42^. The Sn content in the surface Ge_1−*x*_Sn_*x*_ region was calculated to be 12.0% using the normalized areas of the Sn and Ge peaks (Fig. 2c,d), which is close to that in bulk region, and slightly smaller than that in the Ge_1−*x*_Sn_*x*_ oxide layer formed by plasma oxidation. This result indicates negligible Sn atoms migration from inside the Ge_1−*x*_Sn_*x*_ to surface occurs during the plasma oxidation at room temperature.

### Digital Etch of Ge and Ge_1−*x*_Sn_*x*_ layers

**Figure 3:**
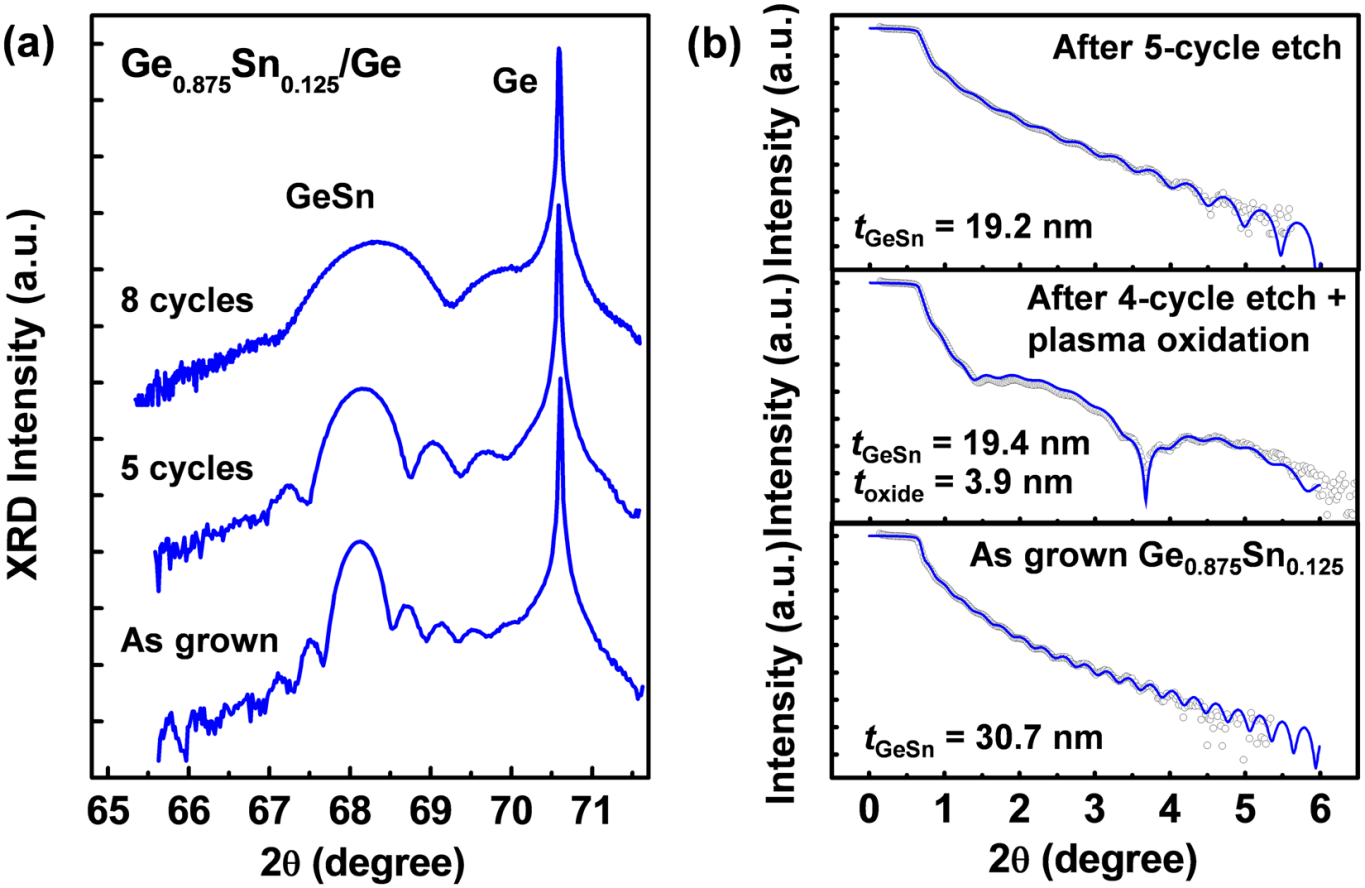
(**a**) (004) HRXRD curves of the as-grown Ge_0.875_Sn_0.125_, and the sample after 5-cycle and 8- cycle digital etch. (**b**) Experimental and simulated XRR curves of the as-grown Ge_0.875_Sn_0.125_, and the sample after 5 cycle plasma oxidation and HCl etch.

High-resolution X-ray diffraction (HRXRD) was used to investigate the Sn content and crystallinity of the Ge_1−*x*_Sn_*x*_ samples. Figure 3a shows the HRXRD scans around the 004 diffraction point for the Ge_0.875_Sn_0.125_/Ge samples before and after digital etch. Clear GeSn and Ge peaks can be identified for the as-grown Ge_0.875_Sn_0.125_ sample, and the presence of interference fringes surrounding the GeSn peak suggests good interface quality. The full width at half maximum (FWHM) of the GeSn diffraction peak increases with increasing etch cycles, which indicates the decrease of Ge_0.875_Sn_0.125_ layer thickness according to Scherrer’s formula^43^. This is consistent with the increasing interval of the interference fringes after digital etch. After an 8-cycle digital etch, the GeSn peak moves slightly towards the Ge peak. The Sn content can be calculated to be 11.7%, which corresponds to a Sn content decrease of approximately 0.8%.

On the other hand, X-ray reflectivity (XRR) measurements have been performed on the Ge_1−*x*_Sn_*x*_ samples before and after digital etch. XRR can be used to determine thickness of thin film with high accuracy by analysing X-ray reflection intensity curves from grazing incident X-ray beam. As a non-destructive technique, no additional lithographic patterning is needed for etch depth calibration. Figure 3b shows the experimental (open black circle) and simulated (blue curve) XRR curves of the as-grown Ge_0.875_Sn_0.125_, the Ge_0.875_Sn_0.125_ after plasma oxidation, and the Ge_0.875_Sn_0.125_ after HCl dipping. The thickness of the as-grown Ge_0.875_Sn_0.125_ film is 30.7 ± 0.2 nm, which can be obtained by fitting the reflected X-ray intensity versus incident angle. The thickness of the Ge_0.875_Sn_0.125_ film decreases with increasing number of digital etch cycles. After a 4-cycle digital etch and plasma oxidation, the thickness of Ge_0.875_Sn_0.125_ film drops to 19.4 ± 0.2 nm. The etch rate can be estimated to be 2.2 nm/cycle. In addition, the thickness of Ge_1−*x*_Sn_*x*_ oxide formed by plasma oxidation can be also extracted by XRR, which is 3.9 ± 0.2 nm. Based on the molar mass and density of Ge and GeO_2_, for every 2.2 nm-thick Ge consumed, 3.97 nm-thick GeO_2_ will appear. This is consistent with the XRR results.

**Figure 4:**
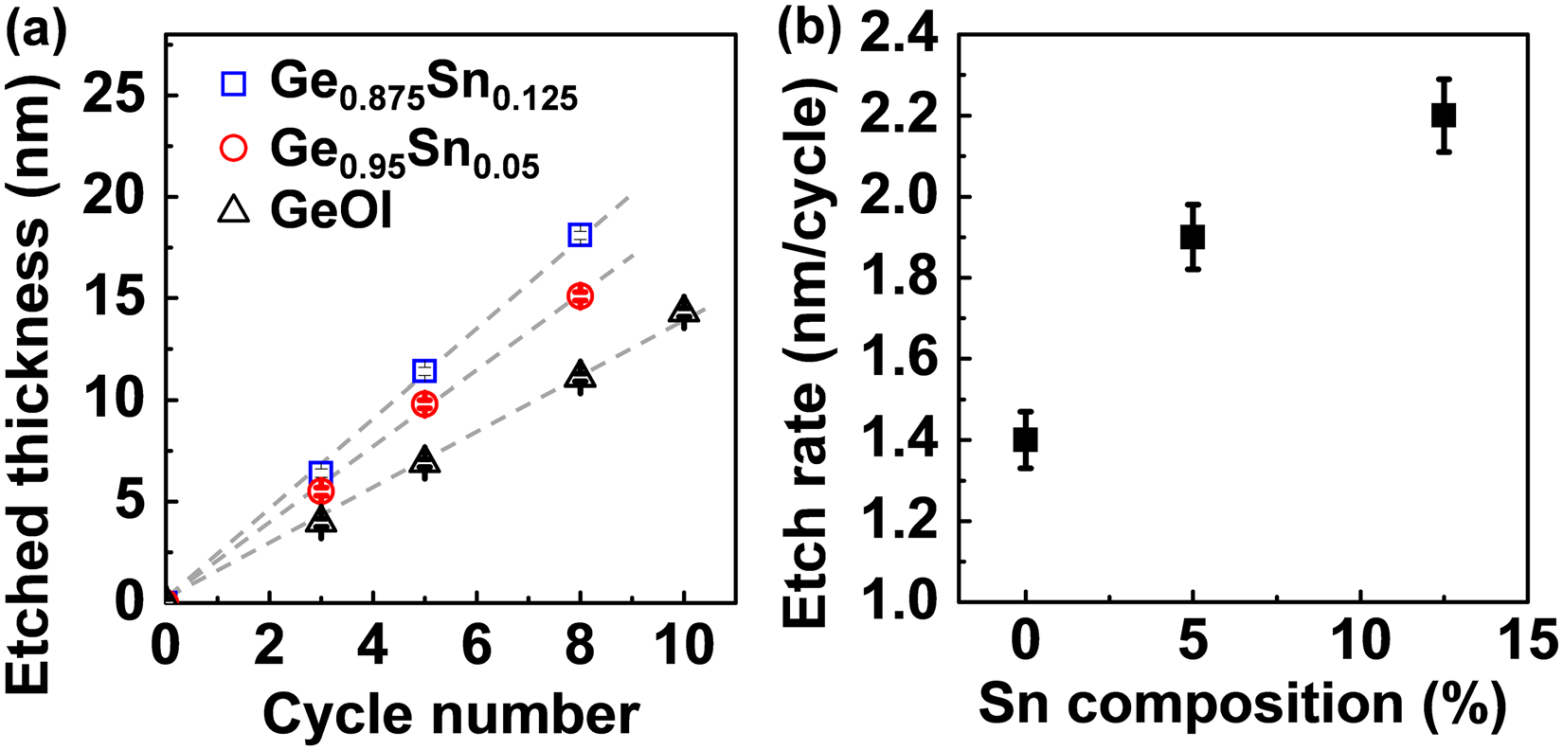
(**a**) Etched thickness versus cycle number with various Sn contents and (**b**) the extracted etch rate. Consistent depth of Ge or Ge_1−*x*_Sn_*x*_ is etched away each cycle.

Figure 4a shows the etched layer thickness as a function of etch cycle number for Ge_1−*x*_Sn_*x*_ samples with various Sn content. The RF power is fixed at 250 W, and the plasma oxidation time is fixed at 120 s. The etched thickness for the Ge_1−*x*_Sn_*x*_ samples was extracted from XRR, while that for the GeOI was obtained from ellipsometry measurements. Good linear fits can be observed in all the samples, which confirm that a consistent depth of Ge or Ge_1−*x*_Sn_*x*_ is etched away each cycle. The etch rate extracted from the slope is ~2.2, ~1.9, and ~1.4 nm/cycle for the Ge_0.875_Sn_0.125_, Ge_0.95_Sn_0.05_, and Ge, respectively (Fig. 4b). It was reported that SiGe alloys had higher oxidation rates than Si under thermal oxidation conditions^44, 45^. This enhancement under thermal oxidation increases with increasing initial Ge concentration in the alloy. Therefore, it could be explained in terms of the Si−Ge bond being weaker than the Si−Si bond. Similarly, the presence of Sn atoms results in weaker Ge-Sn bonds than Ge-Ge bond, and weaker still Sn-Sn bonds at the sample surface (at *T* = 298 K, the dissociation energies for the Ge-Ge, Ge-Sn, and Sn-Sn bonds are 264.4 ± 6.8, 230.1 ± 13, and 187.1 ± 0.3 kJ/mol, respectively)^46^, which may lead to the increased oxidation rate (etch rate) with increasing Sn content. In addition, the introducing Sn may slightly degrade the residual order in the oxide layer, which also affect the etch rate.

**Figure 5:**
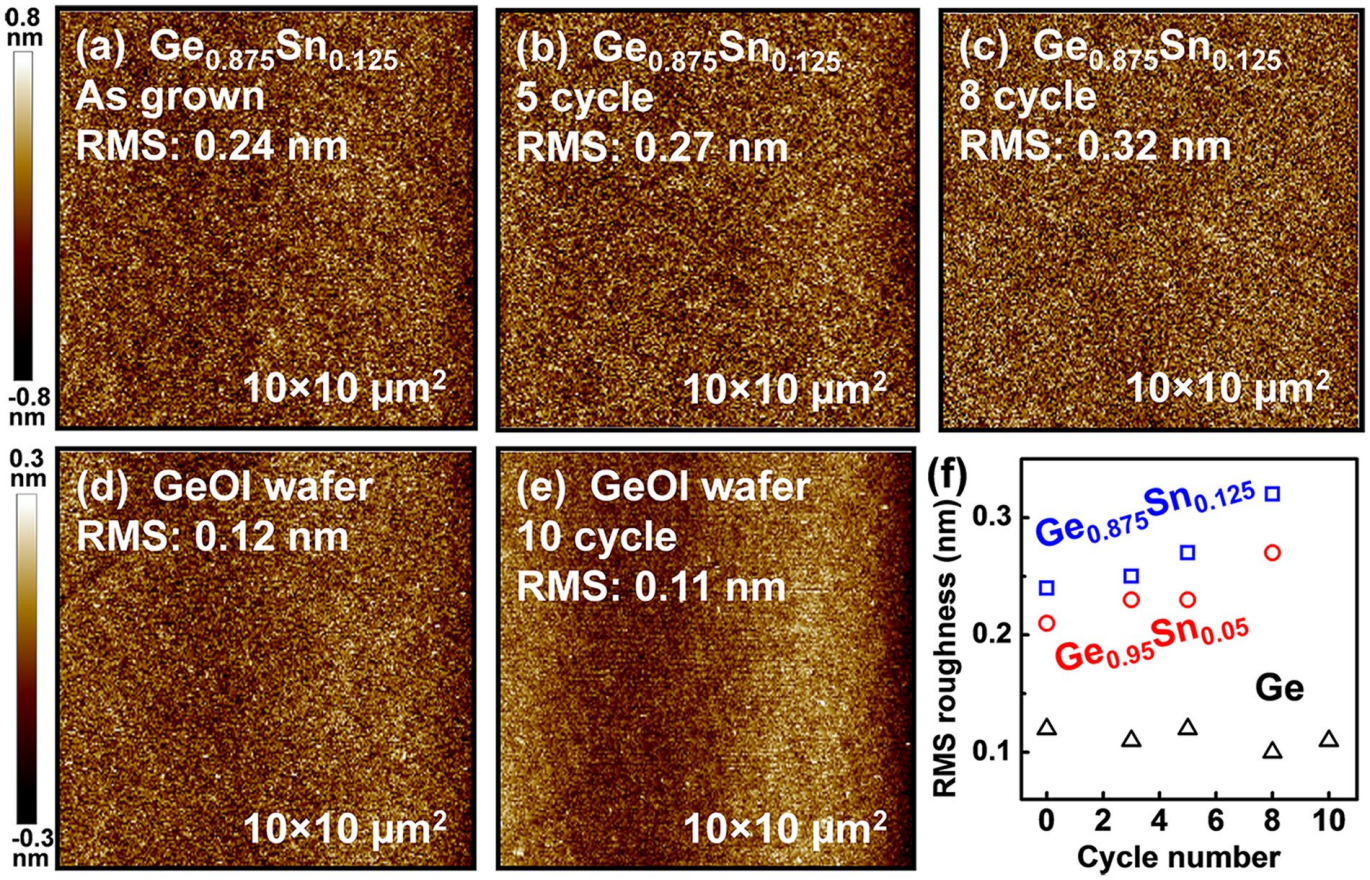
AFM images of (**a**) the as grown Ge_0.875_Sn_0.125_, and the sample after (**b**) 5 cycle and (**c**) 8 cycle digital etch. AFM images of (**d**) the GeOI sample, and the sample after (**e**) 11 cycle digital etch. (**f**) Surface RMS roughness evolution with cycle number. The Ge_1−*x*_Sn_*x*_ surface remains smooth after digital etch.

Surface area to volume ratio increases with downscaling of device dimensions and surface properties can appreciably affect device electronic properties. Atomic force microscope (AFM) was employed to investigate the surface roughness of the Ge or Ge_1−*x*_Sn_*x*_ samples after digital etch. Figure 5a–c show the AFM images for the as-grown Ge_0.875_Sn_0.125_ sample, and the Ge_0.875_Sn_0.125_ samples after 5-cycle and 8-cycle digital etch, respectively. The scan size is 10 × 10 μm^2^, and the z-axis display range of all the images is set to be −0.8 to 0.8 nm, as illustrated on the left side of the figure. The surface remains smooth with ~18 nm-thick Ge_0.875_Sn_0.125_ etched away after 8-cycle digital etch, with the root mean square (RMS) roughness increasing slightly from 0.24 to 0.32 nm. Figure 5d and e show the AFM images of the commercial GeOI wafer and the wafer after 10-cycle digital etch. It was found that the RMS roughness of GeOI samples after etch remains as small as ~0.11 nm. Figure 5f summarizes the RMS roughness of Ge and Ge_1−*x*_Sn_*x*_ samples after various digital etch cycles. The digital etch increases the RMS roughness slightly for Ge_1−*x*_Sn_*x*_ samples but not for GeOI samples. Both GeO_2_ and SnO_2_ were formed on Ge_1−*x*_Sn_*x*_ samples during plasma oxidation. The slightly increased RMS roughness after successive etching cycles may be related to the residual SnO_2_ after HCl dip in which SnO_2_ masks subsequent oxidation, or related to the different oxidation rates for Ge and Sn.

**Figure 6:**
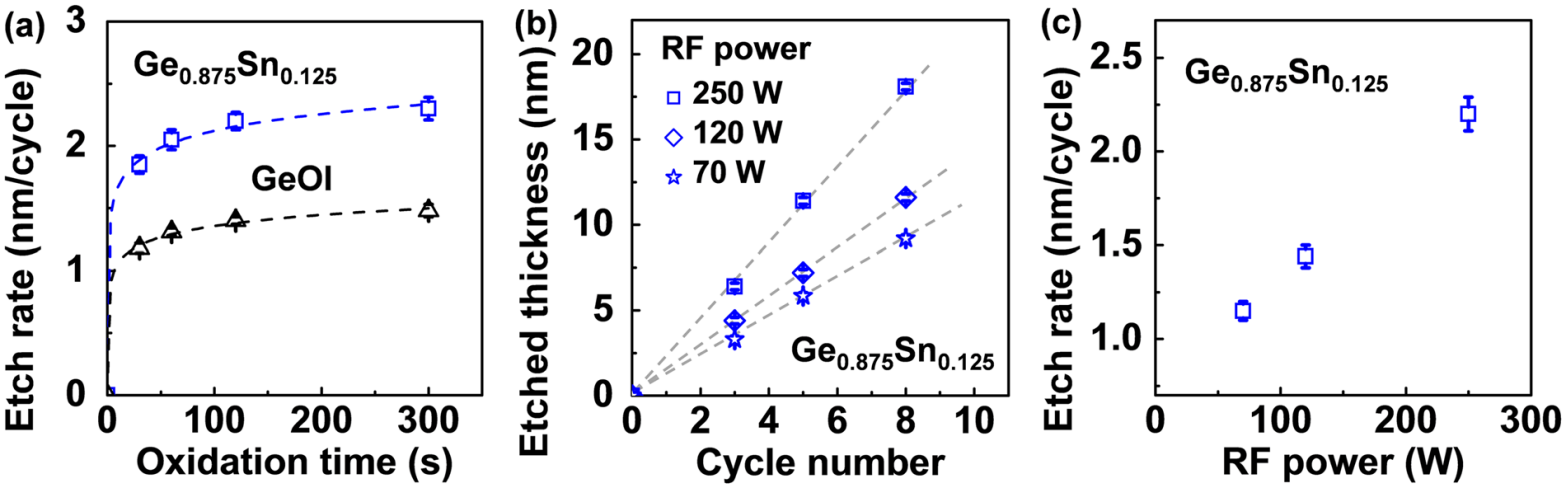
(**a**) Etch rate as a function of oxidation time for Ge and Ge_0.875_Sn_0.125_. The oxide layer thickness grows logarithmically with the oxidation time and saturates fast. (**b**) Etched thickness versus cycle number under different RF power and (**c**) extracted etch rate. The etch rate decreases with decreasing RF power.

Figure 6a shows the measured Ge and Ge_0.875_Sn_0.125_ digital etch rates as a function of plasma oxygen exposure time. The RF power is fixed at 250 W. It is noted that for both the Ge and Ge_0.875_Sn_0.125_ samples, the etch rate increases rapidly for the first 120 s of oxidation time, and then saturates fast to a etch rate of ~1.4 and ~2.2 nm/cycle, respectively. Deal and Grove proposed that the thermal oxidation rate could be determined by a combination of two processes^47^. One is the actual chemical reaction of oxygen O at the interface, and the other is the diffusion of oxygen through the previously formed oxide film. The combination of these processes resulted in the linear-parabolic relationship between the oxide thickness and the oxidation time. However, the Deal-Grove model could not explain the plasma oxidation kinetics well due to the extracted negative value of the reaction rate^48, 49^. K. Kim *et al*. proposed a complementary model of the Deal–Grove oxidation theory to explain the plasma oxidation kinetics, which is involved with the atomic diffusion of O atoms^49^. They found that the oxide layer thickness *y* grows logarithmically with increasing oxidation time *t* as shown below:

1 $$y=\frac{A}{2}\,\mathrm{ln}(\frac{2B(t+\tau )}{{A}^{2}}+1),$$

where *A* and *B* are the parameters related to the effective diffusion coefficient of O atoms in the oxide, and the concentration of O atoms at the oxide surface and in the oxide layer; *τ* is the oxidation time for native oxide formation. This logarithmic limit shows faster saturation of the diffusion-limited process compared with the parabolic limit from the Deal–Grove model. Simply assuming *τ* = 0, good experimental fits for the etch rate (oxide thickness) were obtained with this theory, as shown in Fig. 6a (dash line).

To achieve digital etch with higher precision, the etch rate should be reduced. As discussed, the etch rate can be tuned by varying the oxidation time. However, an ideal digital etch should eliminate the requirement f or timed etching, and decreasing oxidation time may lead to larger variation of etch rate. The etch rate can be also tuned by varying the RF power or gas pressure. Figure 6b shows the etched thickness of Ge_0.875_Sn_0.125_ as a function of etch cycle, and the etch rates were extracted in Fig. 6c. The oxidation time is fixed at 120 s. The etch rate decreases with decreasing RF power, and etch rate of sub-1.2 nm/cycle was obtained with the RF power reduced to 70 W. The main oxidizing species should be atomic oxygen during plasma oxidation^49^. As the atomic oxygen concentration increases with higher RF power, the etch rate increases with higher RF power^49, 50^.

### Formation of Extremely Scaled Ge_1−*x*_Sn_*x*_ Fins

Digital etch technique provides high-precision control of etch, and can be used to realize extremely scale devices, such as deeply scaled FinFETs. According to the 2013 International Technology Roadmap for Semiconductors (ITRS) Overall Roadmap Technology Characteristics (ORTC)^51^, sub-5 nm fin width is required in the sub-10 nm technology nodes. Here we explore the possibility of realizing Ge_1−*x*_Sn_*x*_ fins with 5 nm fin width.

**Figure 7:**
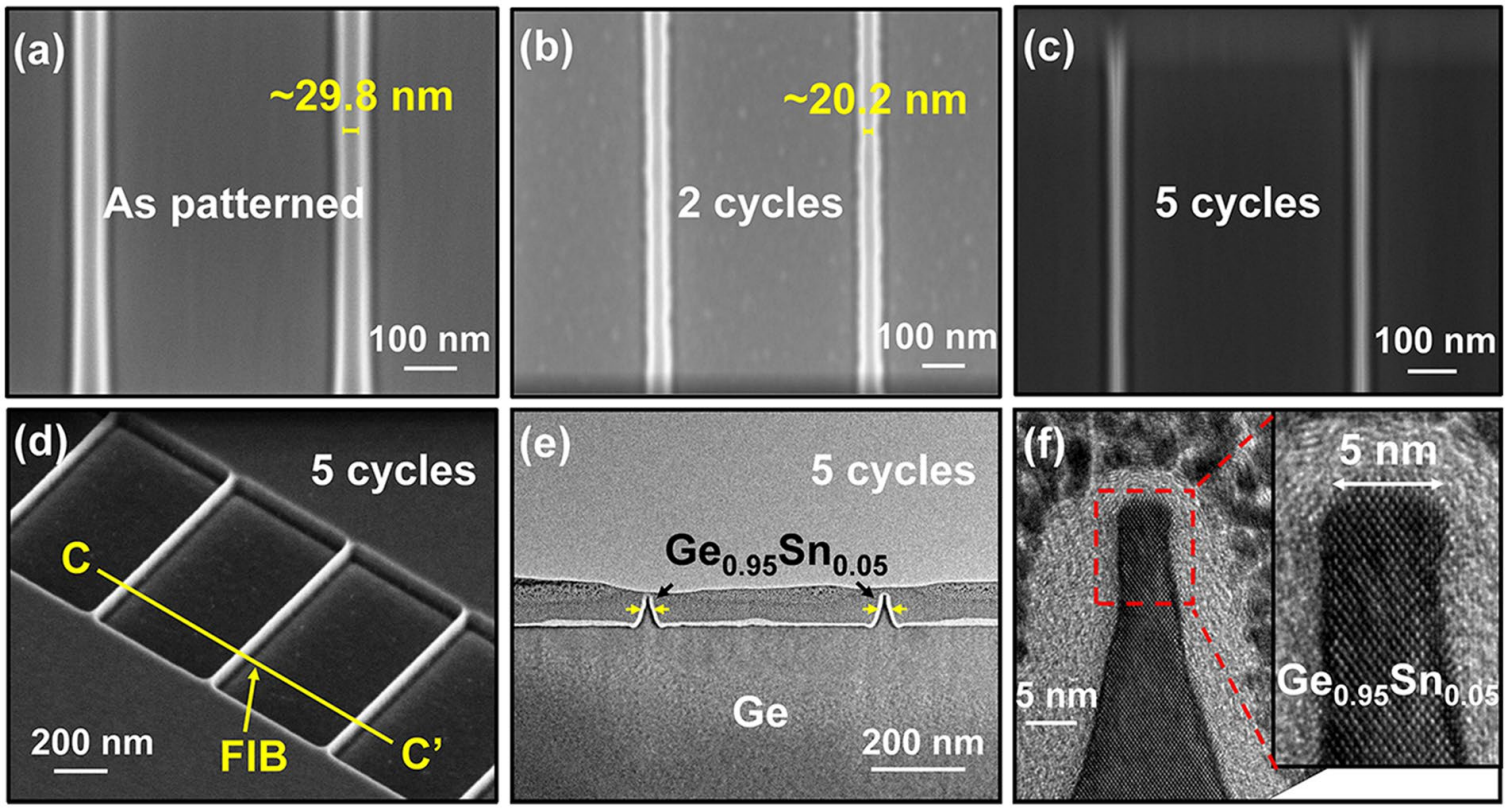
Top view SEM images of (**a**) the Ge_0.95_Sn_0.05_ fins formation by EBL and Cl-based dry etch, and the Ge_0.95_Sn_0.05_ fins after (**b**) 2-cycle and (**c**) 5-cycle digital etch. (**d**) Tilt view SEM image of the Ge_0.95_Sn_0.05_ fins after 5-cycle digital etch. (**e**) XTEM and (**f**) HRTEM images demonstrate high crystalline quality Ge_0.95_Sn_0.05_ fins with fin width of ~5 nm.

The ~28 nm-thick Ge_0.95_Sn_0.05_/Ge sample was used for forming fins. After electron beam lithography (EBL) patterning and chlorine-based dry etch, a trapezoidal Ge_0.95_Sn_0.05_ fin structure was formed, with fin height of ~70 nm and sidewall slope of ~75°. Details of the Ge_0.95_Sn_0.05_ fins fabrication process are provided in the Methods section. Figure 7a shows the top-view scanning electron microscope (SEM) image of the as-patterned Ge_0.95_Sn_0.05_ fins after dry etch. The fin width is ~29.8 nm. Digital etch (RF: 250 W, Oxidation time: 120 s) was performed on the sample, and the fin width obviously shrinks with increased etch cycle (Fig. 7b and c). The etch rate of ~2.4 nm/cycle can be extracted from the SEM image, which is higher than that extracted from bulk Ge_0.95_Sn_0.05_ sample. The different etch rate should arise from different crystal orientation of the etch surface. The patterned Ge_0.95_Sn_0.05_ fins are along <110> direction, thus the oxidation and etch occur on the {110} oriented surfaces. Ligenza *et al*. suggested that the orientation effect on oxidation rate might be caused by differences in the dangling bond density on the various crystal surfaces^52^. As the {100} surfaces have lower dangling bond density than {110} surfaces, the oxidation rate or etch rate for {100} surfaces is slower.

Figure 7d shows the tilted-view SEM image of the Ge_0.95_Sn_0.05_ fins after 5-cycle digital etch. Extremely scaled Ge_0.95_Sn_0.05_ fins were produced with good uniformity. The region where the transmission electron microscope (TEM) lamella was prepared by focused ion beam (FIB) milling is highlighted by a green solid line along CC’. The cross-sectional TEM image of the Ge_0.95_Sn_0.05_ fins is shown in Fig. 7e. Trapezoidal fins with narrow upper base were formed. The high resolution TEM (HRTEM) image of one Ge_0.95_Sn_0.05_ fin is presented in Fig. 7f, demonstrating the high crystalline quality of the Ge_0.95_Sn_0.05_ fins. The fin width was trimmed down to 5 nm after 5-cycle digital etch. The side wall of Ge_0.95_Sn_0.05_ fin is smooth with no observed crystal damage as shown in the inset of Fig. 7f.

In summary, we have demonstrated a new digital etch technique for controllable etching of Ge or Ge_1−*x*_Sn_*x*_ materials with high precision. The two-step digital etch approach consists of Ge_1−*x*_Sn_*x*_ oxide formation by low temperature plasma oxidation and the oxide removal in diluted HCl. The plasma oxidation is a good self-limiting process, as the oxide layer thickness grows logarithmically with the oxidation time and saturates fast. Consistent etch rate can be obtained on the Ge_1−*x*_Sn_*x*_ samples, independent of the initial Sn content. After several cycles of digital etch, the surface morphologies and RMS roughness do not show discernible changes as compared to the as-grown samples. Extremely scaled Ge_0.95_Sn_0.05_ fins with 5 nm fin width were demonstrated with the digital etch technique. The Ge_0.95_Sn_0.05_ fins show high crystalline quality, with smooth side wall. The unique process capability can facilitate fabrication of Ge or Ge_1−*x*_Sn_*x*_ based nanostructures with high-precision control for a range of CMOS, Si-compatible photonics and microelectro-mechanical (MEMS) device applications.

## Methods

### X-ray photoelectron spectroscopy (XPS)

Measurements were performed using a VG ESCALAB 220i-XL imaging XPS system. Monochromatic aluminum (Al) Kα X-ray (1486.7 eV) was employed with the photoelectrons collected at a take-off angle of 90° (with respect to the sample surface). After Shirley-type background subtraction, the normalized peak area is calculated by taking into consideration of the corresponding Scofield photoionization cross-sections (SF), the transmission function of the spectrometer by the manufacturer (TXFN) and the energy compensation factor. The atomic concentration of Sn, *x*, is calculated using

2 $${x}=\frac{{{A}}_{{Sn}}}{{{A}}_{{Sn}}+{{A}}_{{Ge}}},$$

where *A*_Sn_ is the normalized Sn peak area and *A*_Ge_ is the normalized Ge peak area.

### High-resolution X-ray diffractometry (HRXRD)

HRXRD was used to investigate the crystalline quality, composition, and strain of the samples by using the X-ray demonstration and development (XDD) beamline at the Singapore Synchrotron Light Source (SSLS). The wavelength of incident X-ray beam was 0.1634 nm. The diffractometer is the Huber 4-circle system 90000–0216/0, with high-precision 0.0001° step size for omega and two-theta scans. The storage ring, Helios 2, was running at 700 MeV with typical stored electron beam current of 300 mA. X-ray reflectivity measurements were also done at the SSLS.

### Tapping mode atomic force microscope (AFM, Bruker Dimension FastScan)

AFM was employed to characterize the surface morphology and roughness of the samples before and after digital etch. The background slope was removed by subtracting a first order plane fit, and the tilt was removed by a first order flatten with the NanoScope Analysis software.

### Transmission electron microscope (TEM, Tecnai G2 F20 X-Twin)

TEM was employed to investigate the extremely scaled Ge_0.95_Sn_0.05_ fins after digital etch. Specimen for TEM examination was prepared by the Defect Analyzer (DA) 300 Focused Ion Beam (FIB) systems.

### Formation of Ge_0.95_Sn_0.05_ fins

The fins were patterned using a JEOL JBX-6300FS Electron Beam Lithography (EBL) System, with hydrogen silsesquioxane (HSQ) acting as negative tone electron beam resist. The process includes: 1. 2% HSQ spin coat with spin-speed of 3000 rpm; 2. Hot plate bake at 250 °C for 2 minutes; 3. EBL exposure at a base dose of ~8000 μC/cm^2^; 4. Photoresist development of 1 minute using NaOH:NaCl:H_2_O = 1:4:100 solution. 5. Rinse gently with flowing DI water.

After EBL patterning, chlorine-based dry etch was used to form the fin structure by inductively coupled plasma (ICP). Process parameters include ICP power of 100 W, RF power of 100 W, Cl_2_ flow of 50 sccm, pressure of 10 mTorr, and temperature of 25 °C.
